# Discordant Antigenic Properties of Soluble and Virion SARS-CoV-2 Spike Proteins

**DOI:** 10.3390/v16030407

**Published:** 2024-03-06

**Authors:** Sameer Kumar, Souradip Dasgupta, Mohammad M. Sajadi, Greg A. Snyder, Anthony L. DeVico, Krishanu Ray

**Affiliations:** 1 Division of Vaccine Research, Institute of Human Virology, University of Maryland School of Medicine, 725 West Lombard Street, Baltimore, MD 21201, USA; 2 Division of Clinical Research, Institute of Human Virology, University of Maryland School of Medicine, 725 West Lombard Street, Baltimore, MD 21201, USA; 3 Department of Medicine, University of Maryland School of Medicine, 725 West Lombard Street, Baltimore, MD 21201, USA; 4 Department of Biochemistry and Molecular Biology, University of Maryland School of Medicine, 725 West Lombard Street, Baltimore, MD 21201, USA

**Keywords:** fluorescence correlation spectroscopy, SARS-CoV-2 spike, antigenicity, Covi-mAbs, anti-HIV glycan mAbs, in vitro binding assay

## Abstract

Efforts to develop vaccine and immunotherapeutic countermeasures against the COVID-19 pandemic focus on targeting the trimeric spike (S) proteins of SARS-CoV-2. Vaccines and therapeutic design strategies must impart the characteristics of virion S from historical and emerging variants onto practical constructs such as soluble, stabilized trimers. The virus spike is a heterotrimer of two subunits: S1, which includes the receptor binding domain (RBD) that binds the cell surface receptor ACE2, and S2, which mediates membrane fusion. Previous studies suggest that the antigenic, structural, and functional characteristics of virion S may differ from current soluble surrogates. For example, it was reported that certain anti-glycan, HIV-1 neutralizing monoclonal antibodies bind soluble SARS-CoV-2 S but do not neutralize SARS-CoV-2 virions. In this study, we used single-molecule fluorescence correlation spectroscopy (FCS) under physiologically relevant conditions to examine the reactivity of broadly neutralizing and non-neutralizing anti-S human monoclonal antibodies (mAbs) isolated in 2020. Binding efficiency was assessed by FCS with soluble S trimers, pseudoviruses and inactivated wild-type virions representing variants emerging from 2020 to date. Anti-glycan mAbs were tested and compared. We find that both anti-S specific and anti-glycan mAbs exhibit variable but efficient binding to a range of stabilized, soluble trimers. Across mAbs, the efficiencies of soluble S binding were positively correlated with reactivity against inactivated virions but not pseudoviruses. Binding efficiencies with pseudoviruses were generally lower than with soluble S or inactivated virions. Among neutralizing mAbs, potency did not correlate with binding efficiencies on any target. No neutralizing activity was detected with anti-glycan antibodies. Notably, the virion S released from membranes by detergent treatment gained more efficient reactivity with anti-glycan, HIV-neutralizing antibodies but lost reactivity with all anti-S mAbs. Collectively, the FCS binding data suggest that virion surfaces present appreciable amounts of both functional and nonfunctional trimers, with neutralizing anti-S favoring the former structures and non-neutralizing anti-glycan mAbs binding the latter. S released from solubilized virions represents a nonfunctional structure bound by anti-glycan mAbs, while engineered soluble trimers present a composite structure that is broadly reactive with both mAb types. The detection of disparate antigenicity and immunoreactivity profiles in engineered and virion-associated S highlight the value of single-virus analyses in designing future antiviral strategies against SARS-CoV-2.

## 1. Introduction

The COVID-19 global pandemic has claimed at least 7 million lives worldwide, owing to the periodic emergence and circulation of new SARS-CoV-2 variants [1,2,3]. Newer variants seem more transmissible and partially resistant to early vaccines [4,5,6,7] and neutralizing antibodies (NAbs) [8,9,10,11] elicited by early strains. Although vaccines and drugs have reduced infections and deaths, it is highly likely that virus evolution and emergence will be a recurring, worldwide concern for the foreseeable future. Consequently, COVID-19 prevention and treatment must be an ongoing, evolving effort reliant on the ability to understand and counteract genotypic and phenotypic changes that alter SARS-CoV-2 infectivity, transmissibility, and immune escape.

SARS-CoV-2 envelope (Env) spike (S) trimers are critical targets for antiviral countermeasures. These structures mediate virus attachment to and fusion with host cells and are targets for neutralizing antibodies [12,13]. Evolutionary mutations constantly occurring in the human host population modulate both replication functions and immune recognition. On virion surfaces, S is a trimer of two interacting subunits [14], S1 and S2, that mediate attachment to host cells (e.g., human respiratory epithelial cells) via the angiotensin-converting enzyme 2 (ACE2) receptor [15]. S1 contains the N-terminal domain (NTD), the receptor binding domain (RBD), and S1–S2 furin cleavage site. S2 contains an S2 protease site that is cleaved (e.g., by TMPRSS2 or cathepsins) to release its fusion peptide. As with all coronaviruses, the receptor-liganded SARS-CoV-2 trimer transitions through distinct conformational intermediates that “open” the trimer apex and initiate fusogenic activity [16,17,18,19]. Unlike the unliganded S proteins of seasonal human coronaviruses, which are constitutively organized as “closed” trimeric structures [20,21], SARS-CoV-2 trimers variably toggle between closed (all RBDs down toward the virion surface) and “open” (one or more RBDs “up”) conformations [17,18,22,23,24] depending on strain. When the S1 RBD of each S protomer flips to an “up” orientation (pointed away from the virion), the ACE2 receptor is captured [25]. The trimers then transition through additional conformational intermediates that fix the trimer apex in an “open” state with S protomers “up”; shed S1; and place the exposed S2 fusion domain toward the host cell membrane [16,17,18,19,26].

Several lines of evidence associate the emergence and dominance of new SARS-CoV-2 variants with certain mutations in S [27]. Such relationships have been observed since the first stage of the pandemic, when emerging variants with an S1 D614G mutation appeared to have greater transmissibility [28,29]. Subsequently, a host of mutations have been identified with the potential to influence attachment, membrane fusion, and entry functions [30]; confer replication/fitness advantages [31,32,33]; and/or evade immune responses [28,29,34]. The Alpha (B.1.1.7), Beta (B.1.351), and Gamma (P.1) variants express an N501Y mutation in RBD, which could enhance ACE2 binding. The B.1.351 and P.1 strains also include a E484K S mutation that reduces sensitivity to NAbs elicited by earlier variants [35]. Delta (B.1.617.2) variants harbor L452R and P681R mutations, in RBD and near the furin cleavage site, respectively, which also reduce sensitivity to antibody neutralization versus previous variants [31,36,37]. Compared to ancestral (Wuhan) variants, the more recent Omicron BA.1 [38,39] expresses 32 unique amino acid positions in S [40,41]. Notably, the neutralizing activity of serum antibodies from SARS-CoV-2 vaccinees to the ancestral (Wuhan) strain is lower against Omicron subvariants compared to the Wuhan variant [42,43]. As of 2023 [44]; the XBB.1.5 descendant of the BA.2 lineage expresses an F486P S mutation that enhances attachment to ACE2 [45,46]. In view of these findings, it is apparent that S evolution in the human population will be a key barometer for understanding the future course of the COVID-19 pandemic. 

The structure–function relationships of SARS-CoV-2 trimer sequences have been extensively studied using indirect in vitro analyses (e.g., infectivity or neutralization) or the ensemble imaging/computational modeling of static structures [17,18,19,23,24,35,47,48] such as stabilized soluble trimers. Structural analyses of engineered soluble S trimers have been important for understanding how sequence changes alter protein–protein interactions [35,49]. However, such approaches may fail to fully capture the antigenic properties of natural S trimers on virion surfaces. Coarse-grain model simulations of interactions between membrane-bound ACE2 dimers and virion S trimers predicted the disparate structural and functional behaviors of membrane-bound versus soluble S trimers [50]. Further, Förster resonance energy transfer (FRET) imaging of particles pseudotyped with sequence-modified (for fluorophore attachment) SARS-CoV-2 trimers (from a 2020 variant) detected a dynamic structure, exhibiting constitutive fluctuations between four conformational states ranging from all RBD up versus all RBD down protomer positions. The favored state changes as receptor liganding ensues [51]. Caveats of this approach include the need for sequence modifications of S (for placing FRET dye pairs); the presentation of one or a few such modified trimers on a virion; and uncertainties that the modified trimer represents the disposition of all spikes across the entire virion. Nevertheless, evidence of structural dynamics must be considered in the context of virus–NAb or monoclonal antibody (mAb) interactions and immune evasion. Differences between soluble and virion-associated S might explain why certain anti-HIV NAbs are broadly reactive with glycan patches on both HIV gp120 and SARS-CoV-2 S, but neutralize only the former [52,53] and not the latter [52,53]. Such dichotomies warrant further investigation.

Here, we explore, for the first time, the antigenicity of SARS-CoV-2 S and virions in solution, using physiologically relevant mAb concentrations. Our analyses are enabled by fluorescence correlation spectroscopy (FCS), which we successfully applied in order to define the antigenicity of HIV-1 virions with respect to neutralizing and non-neutralizing antibody interactions [54,55,56]. This approach allows direct analyses of unadulterated virus surface proteins at the level of single particles. Immunoreactivity was explored using a panel of non-neutralizing and broadly neutralizing anti-S mAbs, including ones (designated “Covi-series”) we isolated from an infected individual in year 2020 of the pandemic. The epitope targets for these mAbs have been reported [49,57]. These antibodies allowed us to comprehensively track changes in S antigenicity from 2020 forward. A collection of cross-reactive anti-glycan mAbs [58] which neutralize HIV and bind SARS-CoV-2 virions [59,60] were also examined for comparison. Collectively, our data reflect the evolving binding efficiencies of early anti-S mAbs with SARS-CoV-2 variants over time. Importantly, data for individual strains also indicate the antigenic, structural, and/or functional heterogeneity of virion-associated S, which mediates the binding of neutralizing and non-neutralizing mAbs. This heterogeneity is only partially captured by matching soluble S trimers or S-pseudotyped particles.

## 2. Materials and Methods

*Production and labeling of anti-SARS-CoV-2 spike mAbs*. All anti-spike mAbs labeled “Covi” were isolated from donor ID3, who was infected in South Asia in February 2020. The method of isolation, as well as individual mAbs Covi-9, Covi-11, and Covi-17, have been described before [49,57]. In previous publications, Covi-9, Covi-11, and Covi-17 were referred to as CoVIC-78, CoVIC-79, and CoVIC-96, respectively [49], and Covi-11 as Cov11 [57]. Covi-10, Covi-21, and Covi-24 have not been previously reported. CR3022 was obtained from BEI Resources, and 2G12 (#AB002) was purchased from Polymun scientific. PGT121 (ARP12343) and PGT126 (ARP12344) were obtained from HIV reagent program contributed by International AIDS Vaccine Initiative. Fragment of antigen binding (Fab) of 2G12 was generated and purified as described [55]. The nonspecific human IgG1 used as negative control was purchased from Calbiochem, San Diego, CA, USA. All mAbs were fluorescently labeled using Alexa Fluor 647 dye (A647) and purified as described [54]. Briefly, succinimidyl ester moiety of A647 dye reacts with primary amines of the antibody in slightly alkaline condition to form a stable amide bond, producing the dye labeled antibody. The reaction was incubated for 2 h at room temperature. The labeled antibody was purified from free dye by centrifuging in spin column at 1100× *g* for 5 min. The labeled antibodies were characterized by a UV–visible spectrometer (Nanodrop 2000, ThermoFisher Scientific, Wilmington, DE, USA). 

*SARS-CoV-2 recombinant spike proteins and virions.* The following SARS-CoV-2 reagents were obtained through BEI Resources, NIAID, NIH; gamma-irradiated SARS-CoV-2 viruses of ancestral (Wuhan) variant (USA/MD-HP01542/2021) and Lineage B.1.617.2; Delta variant (hCoV-19/USA/MD-HP05285/2021) and full-length SARS-CoV-2 spike protein variants; Alpha (B.1.1.7), Beta (B.1.351), Gamma (P.1), Delta (B.1.617.2), and Omicron (B.1.1.529, BA.2, BA.2.75, BQ.1.1, and BA.4); spike trimer (S1+S2) his-tag (D614G); SARS-CoV-2 perfusion spike ectodomain Hexapro; and recombinant SARS-CoV-2 soluble stabilized spike (Wuhan). The recombinant Wuhan spike protein lacks the signal sequence and contains 1196 residues (ectodomain) of the SARS-CoV-2 spike glycoprotein. It is also modified to remove the polybasic S1/S2 cleavage site (RRAR to A; residues 682 to 685), stabilized with a pair of mutations (K986P and V987P), and includes a thrombin cleavage site, T4 fold on trimerization domain, and C-terminal hexa-histidine tag. Both Wuhan isolate USA/MD-HP01542/2021 and delta isolate hCoV-19/USA/MD-HP05285/2021 virions were gamma-irradiated (5 × 10^6^ RADs) on dry ice, followed by sonication.

RBD sequences were aligned using Clustal Omega [61]. RBD mutations were mapped onto a surface representation of PDB 7A94 using ChimeraX [62]. 

*Cell culture and production of pseudoviruses.* HEK293T and TZM-bl Hela cells were obtained from the American Type Culture Collection (ATCC) and maintained in complete Dulbecco’s modified Eagle’s medium (DMEM) without phenol red. HEK293T cells with stable expressing ACE2 receptor (HEK293T-ACE2) was maintained in complete DMEM. To produce the SARS-CoV-2 pseudovirus, the envelope plasmid (PSF361.2) was constructed first using the spike gene from 2019NCoV S gene (GenScript, Piscataway, NJ, USA) and cloned into pCAGGS expression vector (GenScript, USA). The primers used are as follows: sf1147, CTCTGAATTCGCCATGCCACCATGTTCGTCTTCC; and sf1148, CTCTACCCGGGATCCGATTTAGGTGTAATGC. Furthermore, for better expression, an additional Kozak sequence was added to the N-terminal end. SARS-CoV-2 pseudovirus was generated by co-transfecting the envelope-deficient HIV-1 backbone plasmid, pNL4-3-ΔE-luc (obtained through AIDS Research and Reference Reagent Program, NIAID, NIH, Bethesda, MD, USA), and SARS-CoV-2 spike plasmid (PSF361.2) in HEK293T cells using the FuGENE (Promega, USA) transfection reagents. Cell supernatant was isolated and concentrated using PEG-it (System Biosciences, USA). Quantification of p24 concentration was performed using ELISA kit from R&D system. Control particles lacking envelope (delE) were produced by transfection with pNL4-3-ΔE-EGFP backbone alone as reported earlier [56].

*Fluorescence correlation spectroscopy (FCS).* FCS has been proven to be a valuable method for evaluating virion–mAb interactions at the single-molecule level for several years [54,55,56,63,64,65]. Briefly, a 15 µL reaction containing 3 µg/mL p24 equivalent of SARS-CoV-2 pseudovirus was treated with nonspecific IgG1 (100 µg/mL) for 90 min at 37 °C to block the nonspecific interaction. Subsequently, 1 nM Alexa Fluor 647-conjugated mAb (A647–mAb) was added to the reaction mixture and incubated for 90 min at 37 °C to form immune complexes. Approximately 11 µL of the reaction mixture was loaded onto a glass coverslip sample chamber and sealed before FCS measurements were performed. A 3 µg/mL p24 level of pseudovirus contained approximately 3 × 10^10^ virions/mL. SARS-CoV-2 gamma-irradiated virions (BEI) contained approximately 1 × 10^9^ genome copies/mL, which was further concentrated by ultracentrifugation to 1 × 10^10^ genome copies/mL. FCS reaction conditions rely on limiting protein concentrations that tend to be more physiologically relevant than other reaction systems. Our main goal in configuring reaction conditions was to achieve optimal sensitivity to detect differences in binding efficiency (% antibody bound to antigen) among mAbs. Such conditions occur with limiting amounts of mAb. The reactions we employed (ex: SARS-CoV-2 Wuhan variant) reflect a condition of 3 × 10^10^ particles/mL (pseudovirus) or 1 × 10^10^ particles/mL (inactivated virus) of fluid. Assuming 30 spikes per virion, the overall spike concentration should be ~1.5 nM. Accordingly, we used roughly equimolar (1 nM) concentrations of mAb in the reactions. In the case of purified recombinant SARS-CoV-2 spike protein, a reaction mixture containing 10 nM of spike protein and 5 nM of A647–mAbs was incubated for 30 min at room temperature to form the spike–mAb complexes. As above, 11 µL of this reaction mixture was used for the experiments each time. Different stoichiometric ratios of mAbs to spike protein were used to determine the optimal condition for binding in solution. A molar ratio of 1:2 of mAb to spike protein was selected as it exhibited the maximum percent of binding. FCS measurements were performed in a customized ISS Q2 confocal microscope. The excitation source was Fianium SC-400 super-continuum laser. NKT super-select AOTF filter was used to select the excitation wavelength of 635 nm, which was reflected by a dichroic mirror to an Olympus high-numerical-aperture (NA) water objective (60×; NA 1.2) and focused onto the solution sample. The fluorescence was collected by avalanche photodiodes through a dichroic beam splitter and a band-pass (650–720 nm; Chroma) filter, thus eliminating the scattered excitation light and collecting the fluorescence from the A647-labeled probes in the spectral region of interest. The data acquisition was enabled by a B&H SPC-150 card operated in a photon time-tag time-resolved (TTTR) mode. The recorded data were analyzed using the ISS VistaVision software V4.2 to assess the in vitro binding of A647-labeled mAbs to recombinant spike proteins or SARS-CoV-2 virions. The autocorrelation plots were generated by the VistaVision software and fitted with one-species or two-species 3D-Gaussian diffusion model. The fitting processes yielded the translational diffusion coefficients of A647-labeled mAbs, A647-labeled mAb-bound spike proteins of different variants, and mAb-bound virions. We also determined the fractions of free mAbs, mAbs-bound to SARS-CoV-2 recombinant spike protein, or virions in the reaction mixture.

*Neutralization assay.* The neutralization capability of the Covi-mAbs were assayed using the ACE2 expressing HEK293T cells (HEK293T-ACE2). The SARS-CoV-2 pseudovirus has an HIV-1 backbone containing luciferase gene which, upon infection, can be measured as relative luminescence units (RLUs). For the HIV-1 bnAbs, the neutralizing activity was determined using the TZM-bl target cells [66], which measure Tat-driven luciferase expression after single round of infection. Briefly, 50 µL of SARS-CoV-2 pseudovirus of a TCID_50_ value 20,000 was incubated with 50 µL of 2–7-fold serially diluted test antibodies (starting at 10–50 µg/mL) for 1 h at 37 °C in a CO_2_ incubator. Next, the HEK293T-ACE2 cells (10,000 cells/well) were added to the virus–mAb solution and incubated at 37 °C in 5% CO_2_ incubator. A cell-only control without the virus and a virus-only control without the antibody were included in this assay. After 72 h, Steady-Glo reagent (Promega, Madison, WI, USA) was added and RLU was measured using Victor 3 plate reader (PerkinElmer, Hopkinton, MA, USA) and the percentage of neutralization was calculated.

*Statistical analyses.* Using the GraphPad prism software (Version 10), data were analyzed using either Mann–Whitney’s T-test or the analysis of variance (ANOVA). The statistical significance was defined as *p* < 0.05. 

## 3. Results

### 3.1. Variable Solution Binding of Anti-S mAbs to Recombinant Soluble S from Different SARS-CoV-2 Strains

MAb interactions with soluble S were determined by FCS, adopting procedures we previously used to study the antigenicity of HIV Env. FCS is a sensitive analytical technique that measures fluctuations in the signal intensity of a fluorophore as it passes in and out of a defined focal volume. Such signal fluctuations reflect the fluorophore diffusion kinetic (coefficient), which is a function of mass. Thus, when a fluorophore-labeled molecule binds to a larger target, its diffusion coefficient decreases, owing to the larger mass of the resulting complex. The fraction of labeled molecules assuming a slower diffusion coefficient quantitatively reflects the target binding efficiency. As diffusion coefficients are proportional to mass, FCS measures also validate that fluorescent signals emanate from the expected reaction complexes. For example, a 150 kD free IgG molecule displays a diffusion coefficient of ~55 µm^2^/s; one bound to a 140 kD soluble S protein should diffuse at ~35 µm^2^/s.

To produce a perspective of antigenic changes in variants arising over time, we tested mAbs (e.g., “Covi” series) derived from an individual infected with the earliest known cases of SARS-CoV-2. The panel (Table 1) included neutralizing mAbs Covi-9, Covi-10, Covi-11, Covi-17, and Covi-24, as well as non-neutralizing mAbs such as CR3022 and Covi-21. CR3022 binds to the relatively conserved conformation of the spike protein (amino acids 318–510) in the S1 domain of the SARS-CoV, as well as SARS-CoV-2 strains [67,68]. Members of the panel have been characterized structurally [49,57].

**Figure 1:**
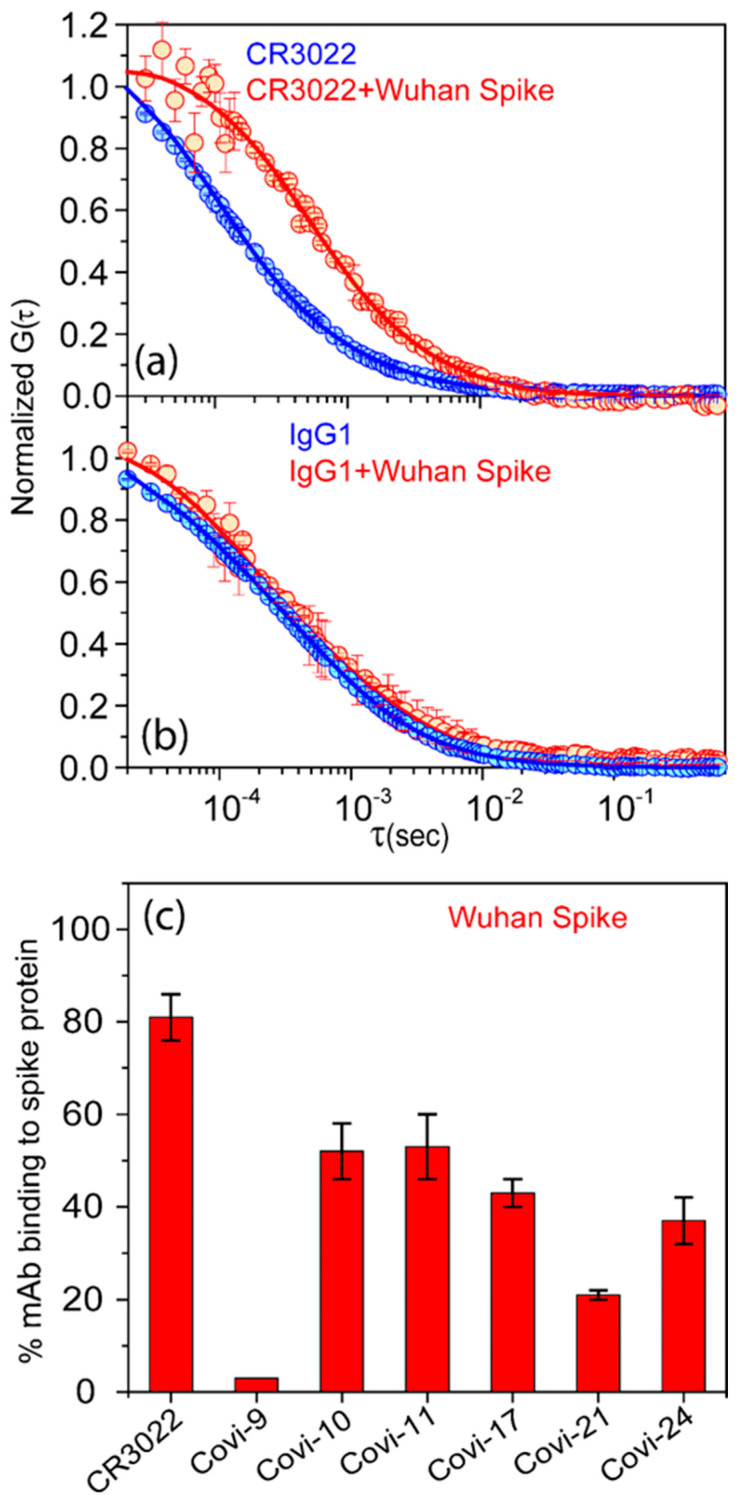
A647-dye-labeled anti-spike mAbs exhibit variable binding efficiencies in reactions with the SARS-CoV-2 spike protein of the Wuhan variant. Representative FCS autocorrelation plots used to calculate binding efficiencies (see text) are shown in panels (**a**,**b**). Antibodies were analyzed with or without exposure to recombinant spike protein. (**a**) mAb CR3022; broadly reactive positive control. (**b**) Non-specific human IgG1; negative control. (**c**) FCS autocorrelation plots such as in panels (**a**,**b**) were used to determine the binding efficiencies (% antibody bound) of various labeled mAbs with the SARS-CoV-2 spike protein. All experiments were performed three times. Data are presented as the mean of three experiments ± sem.

We first tested the mAbs (conjugated to A647 dye) for reactivity with S from the ancestral (Wuhan) variant discovered in Wuhan, China in 2019 [69]. Sequence analysis of the Wuhan variant showed a distinctive polybasic amino acid incorporation in the spike protein that appeared to make it more virulent than other related coronaviruses [70]. MAb CR3022 showed a clear shift in the autocorrelation curve, which fit a two-species diffusion model in which one species exhibited the high diffusion coefficient (55 µm^2^/s) of free mAb and the other showed a lower value of approximately 35 µm^2^/s corresponding to the mAb–S complex (Figure 1a). Applying the FCS-fitting routine (see Methods), we calculated the fractional component of the 35 µm^2^/s species, defining the percentage of the mAb-bound spike in the reaction mixture, i.e., binding efficiency. CR3022 showed a high binding efficiency (approximately 80% bound to S). An irrelevant IgG1 negative control demonstrated only a one-species diffusion rate at 55 µm^2^/s (Figure 1b), indicating no S binding.

When analyzed alone, all other test mAbs displayed auto-correlation plots with single-diffusion coefficients of ~55 µm^2^/s (Supplemental Figure S1). In the presence of soluble Wuhan S, autocorrelation curves consistently fit the two-species diffusion model (one species at 55 µm^2^/s, the other at 35 µm^2^/s) with no other species present. The Covi-series mAbs exhibited a range of binding efficiencies to Wuhan S, ranging from 2% (Covi-9) to 53% (Covi-11) (Figure 1c). Structural studies indicated that Covi-11 Fab binds to the SARS-CoV-2 RBD in a unique orientation, possibly contributing to its neutralization potency. As described earlier, Covi-11 employs a heavy chain VH 3–53 and a light chain VK 3–20 germline sequence towards attaching to the RBD. Most residues of the Covi-11 epitope footprint match with the ACE2-binding site on the RBD [57]. Covi-17 also showed a higher binding efficiency of 43%. Notably, this mAb (coded as CoVIC-096 in [49]) was evaluated by the Coronavirus Immunotherapy Consortium (CoVIC) [71] and determined to have particularly broad and potent neutralizing activity. Negative-stain electron microscopy (NS-EM) characterization suggested that mAb Covi-17 crosslinks two recombinant S proteins [49], possibly explaining the neutralization potency. However, in our reactions, we did not observe species with diffusion coefficients of ~20 µm^2^/sec, indicative of complexes formed by two trimers and two mAbs [49]. The discordance between the NS-EM and FCS data might be explained by differences in reaction conditions. The FCS conditions comprised concentrations of reactants (0.15 µg/mL) mixed in solution at 1:2 molar ratios, whereas, for the NS-EM experiments [49], the complex was formed by incubating the spike and antibody at the same concentrations of 0.25 μg/μL which was equal to a concentration of ~1.6 µM. The FCS experiments were performed at a 1 nM (0.15 µg/mL) concentration of mAbs, which is a ~1600-fold less concentration than the NS-EM experiments. FCS is a sensitive single-molecule-based microscopic technique in which the correlation curve is generated from the fluctuations in fluorescence intensity within the confocal volume in a certain time frame. As FCS is a single (or few)-molecule-based detection technique, based on observations in femtoliter confocal volumes, it cannot be performed at protein concentrations approaching 1 µM. FCS is most sensitive and best performed in the limiting concentration range of 1 to 10 nM. Should a large, cross-linked S protein complex form, FCS would detect it, provided such an object produces a few percent of the total signal events. As we do not observe such a species, we conclude that crosslinking is a phenomenon that mainly occurs in reactions with high concentrations of antigen and antibody. We do not exclude that these interactions are possible. 

**Figure 2:**
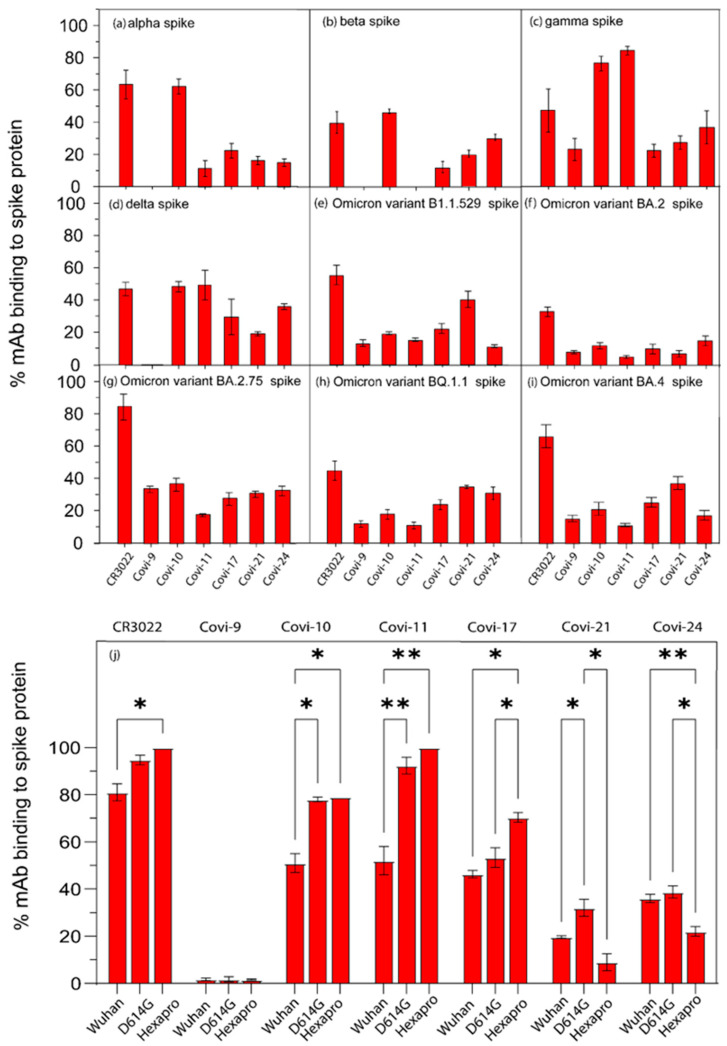
Anti-spike mAbs exhibit variable reactivity with diverse SARS-CoV-2 spike proteins. FCS was used to analyze mAb-binding efficiencies in reactions with the spike proteins of (**a**) Alpha, (**b**) Beta, (**c**) Gamma, (**d**) Delta, (**e**) Omicron (Lineage B.1.1.529), (**f**) Omicron (Lineage BA.2), (**g**) Omicron (Lineage BA.2.75), (**h**) Omicron (Lineage BQ.1.1), and (**i**) Omicron (Lineage BA.4). (**j**) mAb-binding efficiencies in reactions with the spike proteins of Wuhan, D614G mutant, and Hexapro mutant were compared. Data for mAb-binding efficiencies in reactions with the spike proteins of Wuhan variant from Figure 1c was repeated for comparison. Non-specific IgG1 and CR3022 mAb were used as negative and positive controls, respectively. Data are presented as the mean of triplicate measurements ± SEM. Each experiment was performed three times with similar results. * and ** represent *p* values of <0.05 and <0.01 respectively.

SARS-CoV-2 variants that emerged post-(Alpha, Beta, Gamma, Delta, and Omicron) acquired S mutations that alter neutralizing epitopes and/or modulate the constitutive structure of the trimer as an “open” or “closed” structure [72,73,74]. The cross-reactive mAb CR3022 demonstrated substantial immunoreactivity with all these variants (40 to 80% in S-bound form), whereas the negative control IgG1 showed negligible to no evidence of S binding (Figure 2). 

The Alpha variants, first identified in the United Kingdom in 2020 [75], contain an N501Y mutation in S. The Alpha variant tested here showed efficient binding to Covi-10 (62% of the antibody bound to S) but low or no reactivity with any other mAb (Figure 2a). The S protein of Beta variants, first identified in South Africa in 2020, contain a E484K mutation that enables escape from convalescent antisera [75,76]. In this case, only Covi-10 showed substantial immunoreactivity (42% mAb-bound; Figure 2b). Notably, the S trimer of the Gamma variant (Lineage P.1), identified in Brazil in 2020, exhibited broader and more efficient immunoreactivity ranging from 84% (Covi-11) to 23% (Covi-17) mAb-bound (Figure 2c). The S of the Delta variant, first identified in India in 2020, presents several mutations including L452R that reduces sensitivity to neutralizing antibodies elicited by previous strains [75]. Although the Covi mAbs were derived from a subject infected early in the pandemic, four of them (Covi-11, Covi-10, Covi-17, and Covi-24) exhibited binding efficiencies of ≥ 30% mAb attached to S (Figure 2d). The Covi-series mAbs displayed a range of binding efficiencies (15–40 percent complexed to S) across Omicron subvariants (B.1.1.529, BA.2, BA.2.75, BQ.1.1, and BA.4), depending on the reaction mixture (Figure 2e–i). 

We further explored mAb binding to S variants encoding sequence changes that impact structural behavior. The D614G mutation near the S1/S2 boundary of SARS-CoV-2 was reported to allow more open S trimer structures recognized by multiple neutralizing antibodies [29]. Compared to Wuhan S, the D614G mutant was indeed more reactive with Covi-10, 11, 17, 21, and 24, whereas Covi-9 was not bound (Figure 2j). The S1 subunit of the SARS-CoV-2 spike protein is prone to proteases, which results in degradation [77]. The Hexapro spike, based on a soluble Wuhan S, contained six proline substitutions (F817P, A892P, A899P, A942P, K986P, and V987P) that counteract such thermal instability and degradation [78]. Compared to an unmodified soluble Wuhan S, Covi-10, -11, and -17 showed more efficient binding to the Hexapro spike. Nearly all Covi-11 was detected in S-bound form (Figure 2j). In contrast, Covi-9 and 21 showed very weak binding to Hexapro. 

**Figure 3:**
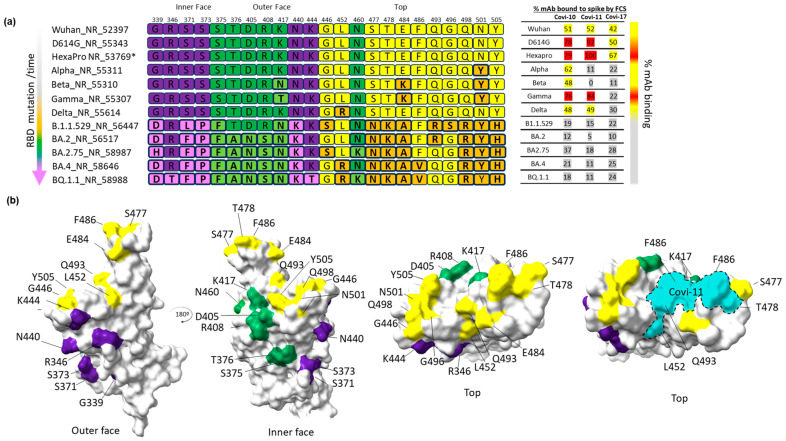
Sequence alignment of variants of concern (VOC) RBD’s mutations and percent binding of mAbs (Covi-10, 11, and 17) to soluble spike protein of different variants. (**a**) Sequence alignment of RBD mutations in VOCs (**left**) compared with the percent mAb binding as measured in solution by FCS (**right**). * Hexapro is a vaccine reagent and is not a VOC. Shown are RBD mutations found on the outer face (purple), inner face (green), and top surface (yellow). VOC RBD mutations deviating from consensus are shown as light purple, light green, and orange. The mAb-binding efficiencies in reactions with the spike proteins of different variants as determined by FCS are color-coded as follows: 75–100% (red), 42–74% (yellow), 0–41% (grey–white). (**b**) Surface representation of RBD mutations. VOC RBD mutations are mapped onto model (PDB:7A94) and colored corresponding to outer (purple), inner (green), and top (yellow) RBD surface views (as previously defined by Callaway et al. [79]). Covi-11-binding epitope [57] on RBD is outlined and shown in cyan (**bottom right**).

The occurrence of mutations in the RBD domain is a conventional explanation for cross-variant differences in mAb-binding efficiencies. This possibility was explored with anti-RBD mAbs Covi-10, -11, and -17, for which the defined epitope and/or structural information is available [49,57]. Figure 3 shows RBD sequences for the listed test variants as they emerged chronologically (Figure 3a), with residue changes mapped onto the respective inner, outer, and top RBD epitope surfaces (Figure 3b) as previously defined by Callaway et al. [79]. The generally lower binding efficiencies with Omicron versus earlier variants is consistent with extensive changes in RBD residue positions, many of which are predicted to establish mAb–RBD contacts [49,57,74,75,79]. In earlier variants (Alpha and Beta), the negligible Covi-11- and Covi-17-binding efficiencies (versus Wuhan) paired with the appearance of mutations at only a single RBD position (417), a predicted mAb contact site. Binding efficiency was higher when mutations occurred elsewhere in the RBD, for example, with Covi-11 and the Beta versus Gamma or Delta strains.

### 3.2. Variable Solution Binding of Anti-Spike mAbs to SARS-CoV-2 Virions

**Figure 4:**
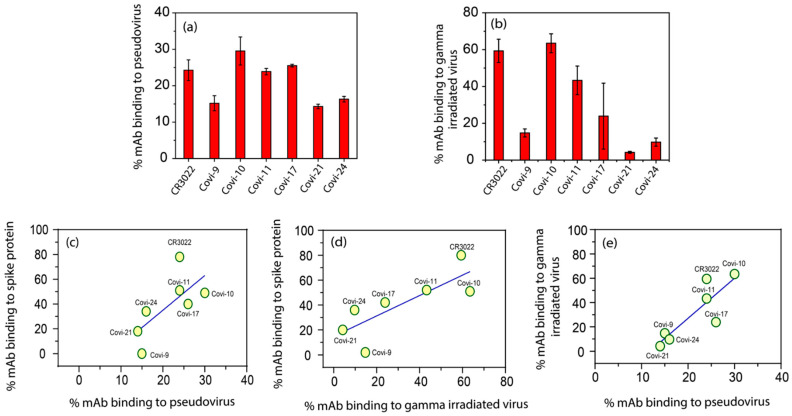
Binding of anti-spike mAbs to SARS-CoV-2 varies between pseudovirions versus gamma-irradiated inactivated virions of Wuhan variant. Autocorrelation plots were used to determine the binding efficiencies (% antibody bound) of a panel of A647-labeled anti-spike mAbs reacted with (**a**) SARS-CoV-2 pseudovirions or (**b**) gamma-irradiated inactivated SARS-CoV-2 virions. Relationships between mAb-binding efficiencies with recombinant Wuhan SARS-CoV-2 spike trimers vs. Wuhan (**c**) pseudovirions (*p* = 0.08 (ns), R^2^ = 0.48) or (**d**) gamma-irradiated virion (*p* = 0.03 (*), R^2^ = 0.62). (**e**) Correlation between mAb-binding efficiencies with pseudovirion vs. gamma-irradiated virion (*p* = 0.01 (*), R^2^ = 0.73). All experiments were performed three times. Data are presented as the mean of three experiments ± SEM.

The above experiments demonstrating variable binding to soluble S trimers prompted us to examine whether similar patterns occur with virus-membrane-associated S. Since FCS is not amenable to the biocontainment analyses of live SARS-CoV-2, the analyses comprised HIV virion-based particles pseudotyped with S trimers, as well as gamma-irradiated (5 × 10^6^ RADs) wild-type virions which retain intact surface structures. We tested two strains, Wuhan (hCoV-19/USA/MD-HP05285/2021) and Delta (hCoV-19/USA/MD-HP05285/2021), for which soluble S trimers, and inactivated viruses with identical RBD sequences are available. In virion reactions, any bound mAb should demonstrate a “slow” diffusion coefficient of ~6 µm^2^/s, corresponding to the ~100 nm size of SARS-CoV-2 virion targets. In accordance with expectations, all virus–mAb reactions produced autocorrelation curves fitting a two-species model; free mAb (55 µm^2^/s) and virion-bound mAb (6 µm^2^/s). As shown in Figure 4a,b, the mAbs again showed a range of binding efficiencies, with both Wuhan pseudoviruses and matched irradiated wild-type virions. In general, the binding efficiencies (percentages of mAbs bound) with virions was lower than what was measured with soluble S (Figure 1). This difference may be explained by higher molar amounts of the target in reactions with the latter. However, there was only a nonsignificant trend toward a direct relationship between mAb-binding efficiencies (percentages of mAbs bound) with the Wuhan pseudovirus versus soluble S trimers (Figure 4c). In comparison, there was a positive and statistically significant (*p* = 0.03) direct relationship between mAb-binding efficiencies with the inactivated virus versus soluble S (Figure 4d). Moreover, there was a significant direct relationship between mAb-binding efficiencies with pseudoviruses versus inactivated wild-type viruses (*p* = 0.01), although the percent mAb-bound was consistently higher with the latter target. Similar results were obtained with the Delta variant targets (Supplemental Figure S2). As shown in Figure S2b, as with the Wuhan variant, there was a statistically significant direct relationship between mAb-binding efficiencies with the inactivated virus versus soluble S (*p* = 0.01) for Delta variants. In control experiments (Supplemental Figure S3), we verified that the test mAbs had negligible to undetectable non-specific interactions with virions by testing delE pseudoviruses, which were produced without an envelope expression construct [56] (see Methods). When mixed with these particles, all mAbs fit only a single-species diffusion model with the signature diffusion coefficient (55 µm^2^/s) of free antibody. 

### 3.3. Neutralizing Capacity of Covi-mAbs against SARS-CoV-2 Pseudoviruses and Correlation of Covi-mAbs Binding Profiles by FCS

**Figure 5:**
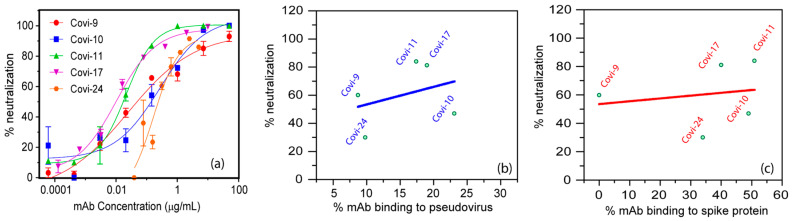
Relationships between mAb-binding efficiency and in vitro neutralization activity against SARS-CoV-2 pseudovirus of Wuhan variant. (**a**) Dose–effect neutralization curves of Covi-series mAbs in assays with the SARS-CoV-2 pseudovirus of Wuhan variant and HEK293T-ACE2 cells (see Methods). Data are presented as the mean of triplicate measurements ± SEM. Relationships between mAb-binding efficiency measured with pseudoviruses (**b**) or recombinant spike protein (**c**) were compared with their neutralization effect at 0.15 µg/mL. Each dot in (**b**) or (**c**) represents a single mAb.

To examine relationships between virion binding and neutralization, the panel of anti-S mAbs were tested in infectivity assays (see Methods) with the Wuhan strain pseudoviruses and HEK293T-ACE2 target cells. As shown in Figure 5a, the mAbs showed a range of neutralization potencies. Covi-17 was the most potent, with an IC50 of 0.01 µg/mL. In agreement with previous reports [80], mAb CR3022 was not neutralizing. We then compared percent virion (Figure 5b) or S binding (Figure 5c) detected by FCS versus percent neutralization at the mAb concentration (0.15 µg/mL) used in the fluorescent binding reaction. Although trends were apparent, there were no statistically significant relationships between binding efficiencies and neutralization potency regardless of whether non-neutralizing mAbs CR3022 and mAb Covi-21 were included in the data (Figure 5b, Supplemental Figure S4). Similarly, there was no significant relationship between neutralization potency and inactivated virion binding (Supplemental Figure S4). For the neutralizing mAbs, we detected IC50 values of 0.01 to 0.21 µg/mL, which equate with ~0.1 to 1.4 nM, which is within the optimum sensitivity range of FCS. Accordingly, FCS binding experiments were performed (Supplementary Figure S5) where mAb concentrations were matched with the IC50 value of the neutralizing antibodies. As in the previous analyses, there were no statistically significant relationships between binding efficiencies and neutralization potency regardless of whether non-neutralizing mAbs CR3022 and Covi-21 were included in the data. The inclusion of mAbs at high molar excess over target would result in a lower % of total antibody in the bound form across the board. This is shown in Supplementary Figure S6, which compares 0.1, 1, and 5 nM of antibodies. Also shown is that, as expected, a low antibody concentration versus target (0.1 nM) reduces signal detection but does not significantly decrease the % antibody bound, i.e., reaction efficiency.

### 3.4. Discordant Binding of Cross-Reactive Anti-Glycan Antibodies to SARS-CoV-2 S Proteins

**Figure 6:**
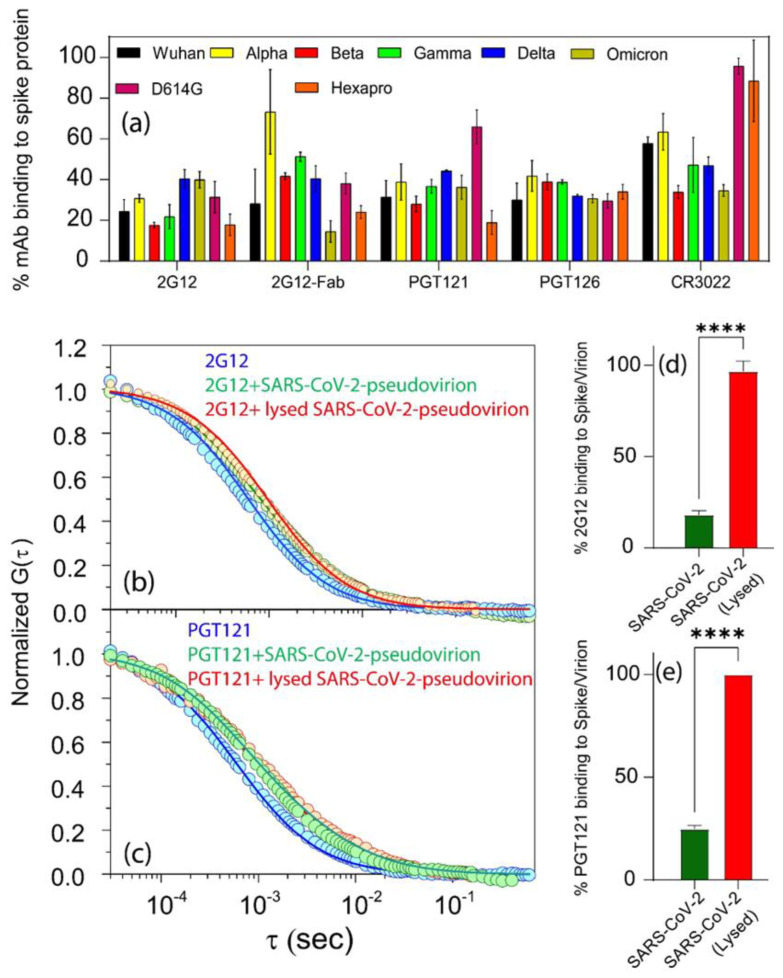
SARS-CoV-2 reactivity with anti-HIV-1 glycan mAbs varies according to the strain and context of spike proteins. In all experiments, CR3022 is included as a positive control and nonspecific human IgG1 as negative control. (**a**) Comparative binding efficiencies of A647-labeled mAbs 2G12, PGT121, PGT126, and Fab 2G12 with soluble spike proteins. mAb 2G12 and PGT121 reactivity was further tested against spike proteins extant on whole pseudovirions or released from particles by detergent lysis. Diffusion coefficients were used to determine the spike protein disposition (virion-bound or released into solution; see text) reactive with the mAb. Fluorescence autocorrelation plots of (**b**) mAb 2G12; or (**c**) mAb PGT121 under the indicated reaction conditions. Autocorrelation plots and diffusion coefficients of signals were used to determine (see text) the binding efficiencies of mAb 2G12 (**d**) or PGT121 (**e**). All experiments were performed three times. Data are presented as the mean of three experiments ± SEM. **** represents a *p* value of <0.0001.

Both HIV-1 and SARS-CoV-2 Env proteins present glycosylated domains and epitopes [81,82]. Antibodies against such epitopes on HIV-1 Env (e.g., 2G12, PGT121, and PGT126) are broadly HIV-neutralizing [83], accordingly termed bnAbs. It has been reported that anti-glycan bnAbs bind soluble S but fail to neutralize SARS-CoV-2 [52,53,84]. In agreement, we failed to detect SARS-CoV-2 neutralization with the antibodies even when tested at up to 10 µg/mL. To elucidate the nature of this dichotomy, FCS was performed using bnAbs 2G12, PGT121, and PGT126 in reactions with S proteins or SARS-CoV-2 pseudoviruses. Similar to the anti-S Covi mAbs, binding efficiencies varied according to virus strain and antibody combinations (Figure 6a). However, in some cases, the bnAbs bound soluble S with efficiencies equal or better than mAb CR3022 (e.g., 2G12, PGT 121, and PGT 126 with Omicron S). Conversely, binding efficiencies with Hexapro were overall lower with bnAbs versus mAb CR3022. To probe whether steric constraints impacted anti-glycan bnAb binding to soluble trimers, the binding efficiency of a smaller Fab fragment of 2G12 was compared with whole IgG. Higher binding efficiencies of Fab vs. whole IgG were apparent and statistically significant for reactions with Alpha (*p* < 0.001), and Gamma (*p* < 0.01) S proteins but not with other S variants (Figure 6a). The Wuhan strain pseudovirus was used as a test case to examine anti-glycan bnAb binding to virions. All bnAbs showed low binding efficiencies (Supplemental Figure S7), equivalent to those of Covi-neutralizing and non-neutralizing mAbs and CR3022. Notably, 2G12 Fab was not significantly more reactive with virions compared to whole IgG, suggesting more extreme steric constraints at the virion surface than occurs with soluble trimers. 

We next examined whether anti-glycan bnAb binding to virions is limited by the nature of the S trimer presentation in the virus membrane. Accordingly, we used anti-glycan bnAbs 2G12 and PGT121 to probe the Wuhan pseudovirions, treated or untreated by detergent to release free S trimers (see Methods). MAb CR3022 was tested as a control. As shown in Figure 6b,c, with untreated/intact virions, the anti-glycan bnAbs showed low binding efficiencies consistent with the previous experiments. In comparison, detergent treatment produced a remarkably different binding profile. The autocorrelation curves fit a two-species diffusion model, but one comprising only free IgG and a mAb-bound species with a diffusion coefficient (35 µm^2^/s) matching the predicted size of a mAb-free trimer complex. No residual virion binding was evident. Complexes of antibody and dissociated S protomers, which would display diffusion coefficients of ~45 µm^2^/sec, were not observed. Most importantly, both 2G12 and PGT121 demonstrated extremely high binding efficiencies with the released S trimers (97% and 100%, respectively; Figure 6d,e), which were otherwise poorly or not reactive with the anti-S mAbs (Supplemental Figure S8). 

## 4. Discussion 

Soluble SARS-CoV-2 S proteins will be a key point of focus in COVID-19 vaccine design for the foreseeable future. Non-pathogenic S-pseudotyped viruses will continue to support assessments of humoral neutralizing antibody activity and other antiviral immune functions. Therefore, the elucidation of antigenic differences between these reagents versus native virions will inform the refinement of immunotherapeutic and/or vaccine countermeasures against COVID-19. Towards this end, here, we utilized a single-molecule-based analytical approach to define antigenic differences in unalloyed soluble SARS-CoV-2 spike proteins, pseudovirions, or inactivated virions representing virus variants emerging over the course of the COVID-19 pandemic. 

We used test mAbs isolated early in the pandemic to elucidate any trends in antigenic changes in variants emerging over time. All test mAbs except Covi-9 demonstrated binding to soluble S trimers from the contemporaneous Wuhan strain, but with variable efficiency (Figure 1c and Supplemental Figure S1). Notably, the non-neutralizing mAb CR3022 [80] demonstrated the most efficient binding. As expected, each mAb demonstrated variable anti-S binding efficiencies across variants (Figure 2) that emerged after the Wuhan strain. Except for CR3022, mAb-binding efficiencies trended lower for more recently emerging (e.g., Omicron) strains. A variant of the Wuhan strain with the D614G mutation, which increases transmissibility and cell entry [27], showed a higher binding efficiency to Covi-10 and Covi-11 compared to the parental virus (Figure 2j). The Hexapro spike, which has six proline substitutions in the Wuhan strain that enable higher expression and stability, and a prefusion S conformation [78], also demonstrated more efficient binding to Covi-10 and Covi-11 but showed less efficient binding to Covi-21 and Covi-24. The latter data indicate that structural flexibility might be an important factor in mAb binding.

The appearance of evolutionary changes in RBD target epitopes were associated with changes in mAb-binding efficiency. For the three mAbs (Covi-10, 11, and 17) evaluated in this context, binding efficiencies trended downward with more recently occurring strains in concert with the accumulation of point mutations in the RBD (Figure 3). Covi-11 was an interesting case where the binding efficiency was severely reduced in the Alpha and Beta strains due to mutations at contact position 417, but was retained for Gamma in concert with other mutations at positions 493 and 498 in the RBD (Figure 3b) [57]. Thus, immune evasion from some mAbs may not be fixed in time, and instead appear and disappear according to other selective pressures on S that drive sequence changes.

Notably, binding patterns and efficiencies across the panel of mAbs also varied according to whether S was presented on a soluble trimer, pseudovirus, or inactivated wild-type virion (Figure 1, Figure 2, and Figure 4) with the same epitope sequences. There were trends but no statistically significant relationships between various mAb-binding efficiencies on soluble trimers versus pseudoviruses (Figure 4c) but significant direct relationships between binding efficiencies on inactivated virions versus pseudovirions or soluble S (Figure 4e,d). For individual mAbs the comparatively low percent binding on pseudoviruses was likely due to lower levels of surface S trimer versus wild-type virions. Notably, no significant relationships appeared between the neutralization potency versus binding to any target. These data indicate that S antigenicity varies according to its mode of expression and that biochemical variables other than the overall virion binding efficiency impact the anti-S neutralization capacity (see below). 

Insights into the lack of a correlation between binding efficiency and neutralizing activity were provided by analyses of HIV-neutralizing bnAbs that cross-react with SARS-CoV-2 S via their specificity for S glycan clusters [52,59,85,86,87,88,89]. Using our in vitro single-molecule fluorescence-based solution binding approach, we observed variable cross-strain binding of such bnAbs (2G12, PGT121, and PGT126) to soluble S proteins and pseudoviruses (Figure 6) but no neutralizing activity, in accordance with previous reports [52,53]. The relatively lower binding efficiencies with pseudoviruses (Figure 6b–e, Supplemental Figure S7) was not readily tracked to steric hindrance on virion surfaces, as the binding efficiency of 2G12 Fab was not significantly higher than whole IgG (Supplemental Figure S7). However, the detergent release of S trimers from virion membranes efficiently bound bnAbs (Figure 6d,e) but poorly reacted with Covi mAbs (Supplemental Figure S8), indicating that they assume a nonfunctional structure that, nevertheless, retains and, perhaps, optimizes the glycan presentation patterns recognized by the bnAbs.

Collectively, the FCS data are consistent with a scenario in which mAb binding to SARS-CoV-2 virions depends not only on the epitope sequence but also factors that modulate the known structural flexibility of S trimers [49,74,90,91]. We consider one heuristic model where virion trimers asynchronously swing between two end states, one functional and the other nonfunctional. The former is recognized by neutralizing antibodies (thus allowing them to neutralize) and the latter by anti-glycan mAbs, which are not antiviral by virtue of targeting a nonfunctional structure. It is possible that a certain fraction of spikes become locked in the nonfunctional form, depending on the virus or method of virion production. Thus, virions of a given strain can be antigenic for both neutralizing (e.g., Covi) and non-neutralizing antibodies (e.g., anti-glycan), with moderate binding efficiencies. It is likely that certain neutralizing mAbs also recognize structural intermediates between the two end states. Such fine preferences, perhaps combined with differences in S-binding affinities, could further explain why virion-neutralizing activity does not correlate with mAb-binding efficiency. In any case, our data suggest that the release of native S from virions causes a broad conversion to the nonfunctional state, improving anti-glycan mAb binding and abrogating reactivity with neutralizing anti-S antibodies. Stabilized soluble S trimers may represent a fixed intermediate in the virion S structural repertoire, being recognized by neutralizing mAbs, non-neutralizing anti-S mAbs, and anti-glycan Abs. This property would explain why soluble-S-binding- versus pseudovirion-binding efficiencies are not correlated. 

## 5. Conclusions

Three main conclusions can be drawn from the FCS data, which reflect interactions between unperturbed reactants at physiologically relevant concentrations. First, FCS is sufficiently sensitive for differentiating anti-S mAb-binding efficiencies according to variants with altered target epitope sequences. Importantly, FCS measures reflected that mAbs elicited by an early SARS-CoV-2 strain demonstrate reduced FCS binding efficiencies with more recently emerging strains. Second, S trimers for a given variant display discernable differences in antigenic properties and immunoreactivity according to whether they are expressed as soluble or virion-associated molecules. Thus, immunochemical analyses of mAbs with soluble S might over- or underestimate reactivity with virions. Soluble S trimers are undoubtedly valuable for studies of anti-SARS-CoV-2 humoral immunity and vaccine development. Continued efforts to develop versions that more closely mirror the characteristics of virion trimers should be useful in refining vaccine and immunotherapeutic designs. Analytical approaches such as FCS can aid such efforts. Third, the structural flexibility and consequent heterogeneity of S trimers likely allow virion immunoreactivity with both neutralizing and non-neutralizing anti-S or anti-glycan antibodies. The possibility that non-neutralizing mechanisms of humoral immunity (e.g., ADCC) against SARS-CoV-2 occur via the antibody recognition of nonfunctional S structures merits further investigation.

## Figures and Tables

**Table 1:** mAbs selected for the current study.

Antibody	Specificity
CR3022	anti-spike S1 (RBD 7)—non-neutralizing
Covi-9	anti-RBD—neutralizing (RBD 5)
Covi-10	anti-RBD—neutralizing
Covi-11	anti-RBD—neutralizing (RBD 2)
Covi-17	anti-spike—neutralizing (RBD 5)
Covi-21	non-neutralizing
Covi-24	anti-spike—neutralizing
2G12	anti-HIV Env, targets glycan patch
PGT-121	anti-HIV Env, targets V3 glycan
PGT-126	anti-HIV Env, targets V3 glycan
Non-specific IgG1	negative control

## Data Availability

All data are included in the main manuscript and supplementary figures.

## Supplementary Materials

The following supporting information can be downloaded at: https://www.mdpi.com/article/10.3390/v16030407/s1, Figure S1: Anti-spike mAbs binding to SARS-CoV-2 spike protein of the Wuhan variant; Figure S2: Binding of anti-spike mAbs to gamma irradiated inactivated virions of Delta variant.; Figure S3: Non-specific binding of anti-spike mAbs to virions of lacking any Env protein; Figure S4: Relationships between Covi-series mAbs binding efficiency and neutralization; Figure S5: Binding of anti-spike mAbs to SARS-CoV-2 pseudovirions of Wuhan variant; Figure S6: Binding of anti-spike mAbs to SARS-CoV-2 pseudovirions of Wuhan variant at varied concentration range; Figure S7: Comparative binding efficiencies of mAbs 2G12, PGT121, PGT126, CR3022 and Fab 2G12 with SARS-CoV-2 pseudovirion of Wuhan variant; Figure S8: MAb CR3022 and Covi- mAbs reactivity against spike proteins extant on whole pseudovirions or released from particles by detergent lysis.
