# Supplementary material for: Discordant Antigenic Properties of Soluble and Virion SARS-CoV-2 Spike Proteins
Source: Viruses. 2024 Mar 6;16(3):407. doi: 10.3390/v16030407 (PMC10974403; doi:10.3390/v16030407)
Supplement: Supplementary file 1 [file viruses-16-00407-s001.zip › viruses-2857540-supplementary.pdf]

viruses

MDPI

Supplementary Figures

Figure S1. A647 dye labeled anti-spike Covi-series mAbs exhibit variable binding efficiencies in reactions with the SARS-CoV-2 spike protein of the Wuhan variant. Representative FCS autocorrelation plots used to calculate binding efficiencies (see text) are shown in panels. All experiments were performed three times.

Figure S2. (a) Binding of anti-spike mAbs to gamma irradiated inactivated virions of Delta variant. Two-species fit to the autocorrelation plots were used to determine the binding efficiencies (% antibody bound) of a panel of A647 labeled anti-spike mAbs reacted with gamma irradiated inactivated SARS-CoV-2 virions (Delta variant). All experiments were performed three times. Data are presented as the mean of three experiments ± SEM. (b) Relationships between mAb binding efficiencies with recombinant SARS-CoV-2 Delta spike trimers vs gamma irradiated virions of Delta variant ($p = 0.01$ (*), $R^2 = 0.8$).

Figure S3. Non-specific binding of anti-spike mAbs to virions of lacking any Env protein (ΔEnv) were examined. Two-species fit to the autocorrelation plots were used to determine the binding efficiencies (% antibody bound) of a panel of A647 labeled anti-spike mAbs reacted with ΔEnv virions. All experiments were performed three times. Data are presented as the mean of three experiments ± SEM.

Figure S4. Relationships between Covi-series mAb binding efficiency measured with Wuhan pseudovirions (a), recombinant Wuhan spike protein (b) and gamma irradiated inactivated Wuhan SARS-CoV-2 virions (c) were compared with their neutralization effect at 0.15μg/ml. Each dot in the panels represents a single mAb. In the panels (a), (b) and (c), same data as in Figure 4 for the neutralizing mAbs are presented, however the binding efficiencies of the non-neutralizing mAbs to S or virions are also included.

Figure S5. (a) Binding of anti-spike mAbs to SARS-CoV-2 pseudovirions of Wuhan variant. IC50 concentrations of neutralizing mAbs were used for FCS binding studies. The concentrations of non-netralizing mAbs CR3022 and Covi-21 were 0.015 μg/ml. Autocorrelation plots were used to determine the binding efficiencies (% antibody bound) of a panel of A647 labeled anti-spike mAbs reacted with SARS-CoV-2 pseudovirions. (b) Relationships between Covi-series mAb binding efficiency measured with Wuhan pseudovirions were compared with their neutralization effect.

Figure S6. Binding of anti-spike mAbs to SARS-CoV-2 pseudovirions of Wuhan variant at the concentration range of 0.1 nM to 5nM. Autocorrelation plots were used to determine the binding efficiencies (% antibody bound) of A647 labeled anti-spike mAbs reacted with SARS-CoV-2 pseudovirions.

Figure S7. Comparative binding efficiencies of A647 labeled mAbs 2G12, PGT121, PGT126, CR3022 and Fab 2G12 with SARS-CoV-2 pseudovirion of Wuhan variant. All experiments were performed three times. Data are presented as the mean of three experiments ± SEM.

Figure S8. MAb CR3022 and Covi-series mAbs reactivity was further tested against spike proteins extant on whole pseudovirions or released from particles by detergent lysis. Diffusion coefficients were used to determine the spike protein disposition (virion-bound or released into solution; see text) reactive with the mAb. Autocorrelation plots and diffusion coefficients of signals were used to determine (see text) the efficiencies of CR3022 and Covi-series mAbs binding to pseudovirions or lysed virions. All experiments were performed three times. Data are presented as the mean of three experiments ± SEM. * ($p < 0.05$), ** ($p < 0.01$), *** ($p < 0.001$), and **** ($p < 0.0001$).

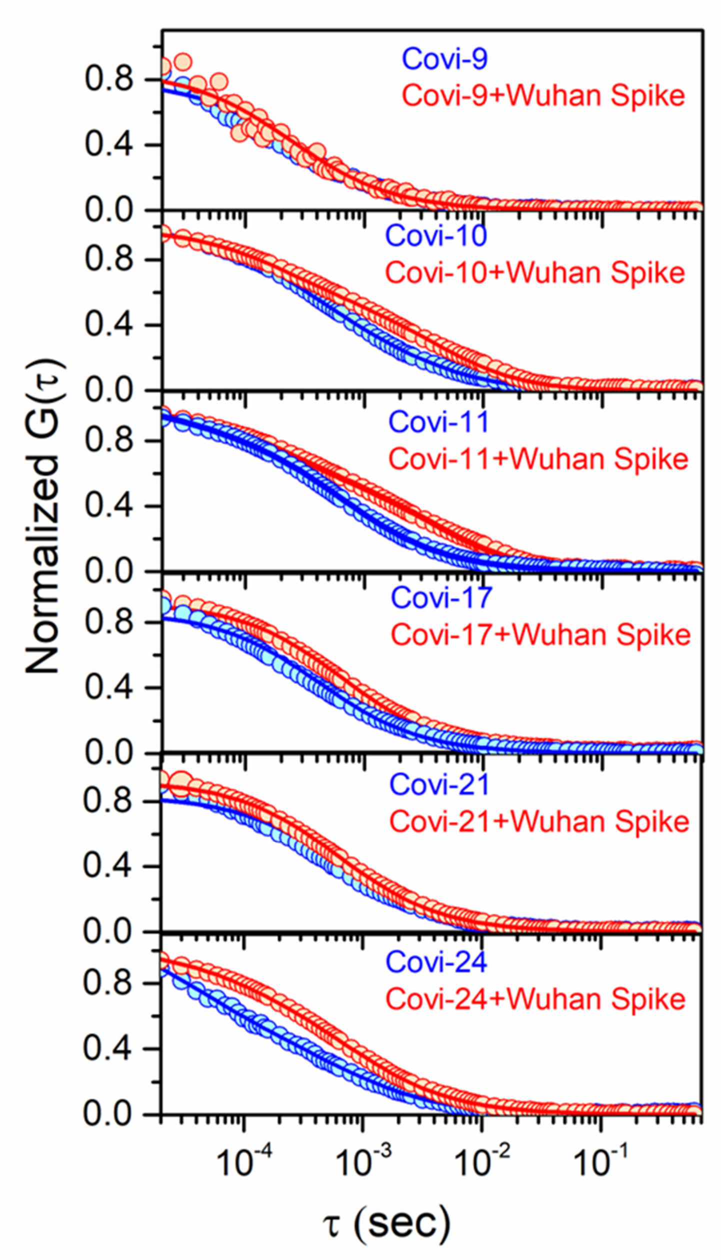

Figure S1. A647 dye labeled anti-spike Covi-series mAbs exhibit variable binding efficiencies in reactions with the SARS-CoV-2 spike protein of the Wuhan variant. Representative FCS autocorrelation plots used to calculate binding efficiencies (see text) are shown in panels. All experiments were performed three times.

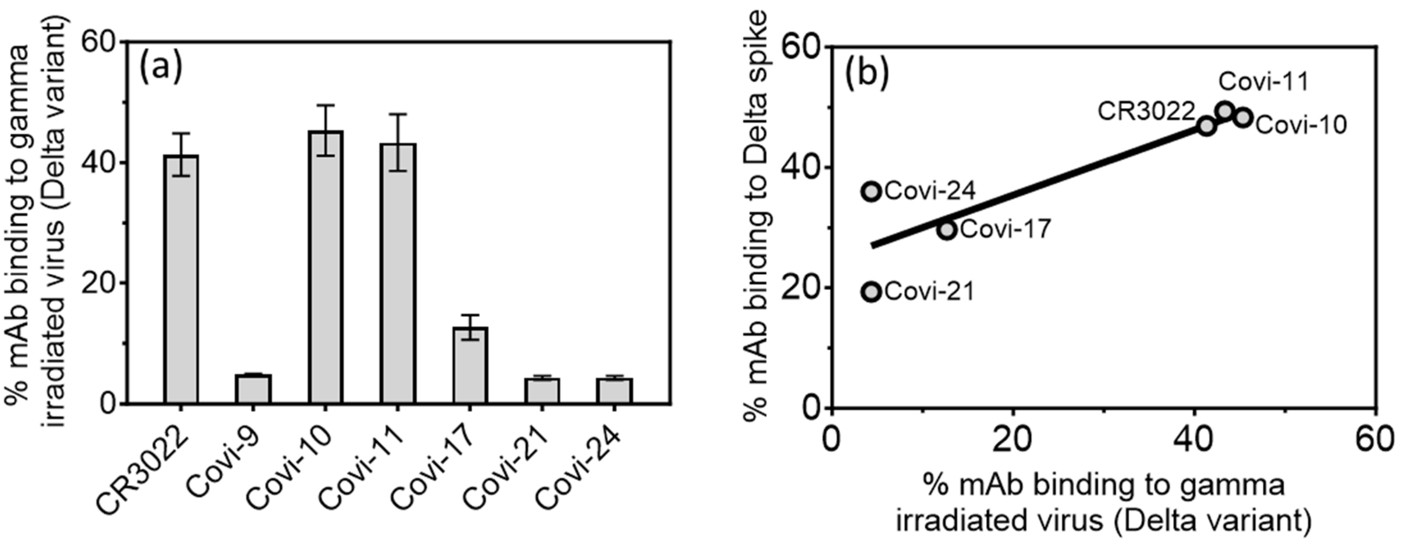

Figure S2. (a) Binding of anti-spike mAbs to gamma irradiated inactivated virions of Delta variant. Two-species fit to the autocorrelation plots were used to determine the binding efficiencies (% antibody bound) of a panel of A647 labeled anti-spike mAbs reacted with gamma irradiated inactivated SARS-CoV-2 virions (Delta variant). All experiments were performed three times. Data are presented as the mean of three experiments ± SEM. (b) Relationships between mAb binding efficiencies with recombinant SARS-CoV-2 Delta spike trimers vs gamma irradiated virions of Delta variant ($p = 0.01$ (*), $R^2 = 0.8$).

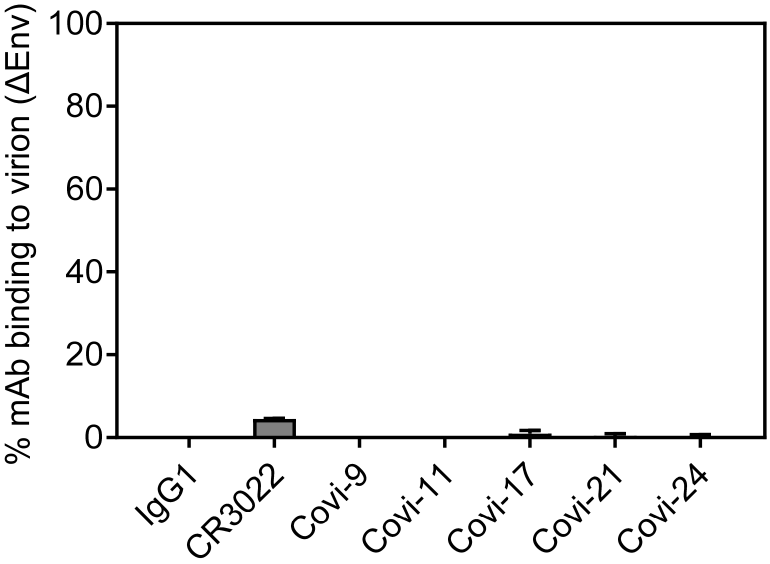

Figure S3. Non-specific binding of anti-spike mAbs to virions of lacking any Env protein (ΔEnv) were examined. Two-species fit to the autocorrelation plots were used to determine the binding efficiencies (% antibody bound) of a panel of A647 labeled anti-spike mAbs reacted with ΔEnv virions. All experiments were performed three times. Data are presented as the mean of three experiments ± SEM.

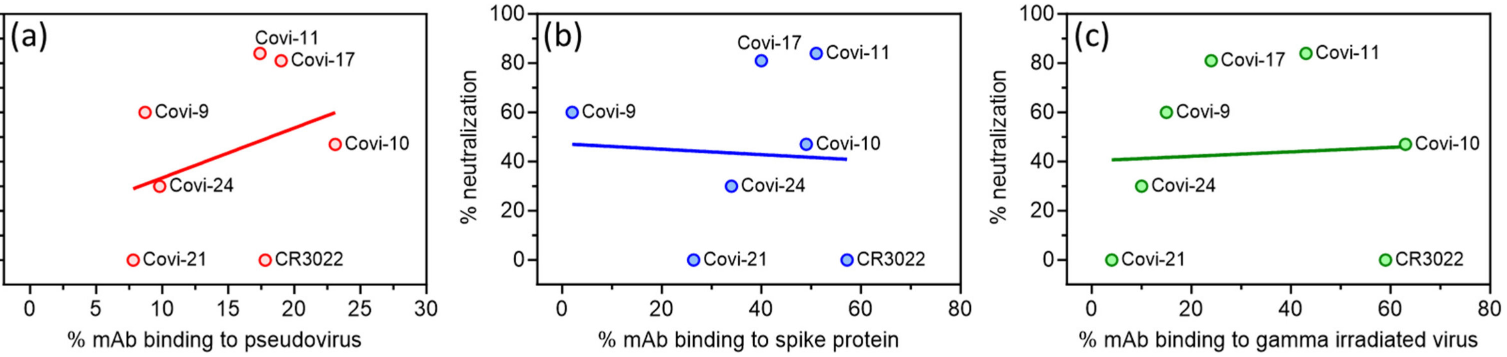

Figure S4. Relationships between Covi-series mAb binding efficiency measured with Wuhan pseudovirions (a), recombinant Wuhan spike protein (b) and gamma irradiated inactivated Wuhan SARS-CoV-2 virions (c) were compared with their neutralization effect at 0.15µg/ml. Each dot in the panels represents a single mAb. In the panels (a), (b) and (c), same data as in Figure 4 for the neutralizing mAbs are presented, however the binding efficiencies of the non-neutralizing mAbs to S or virions are also included.

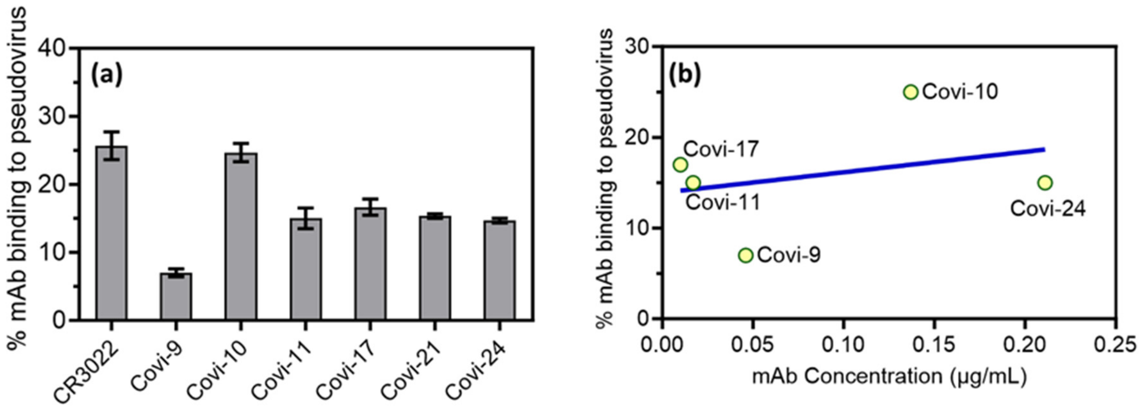

Figure S5. (a) Binding of anti-spike mAbs to SARS-CoV-2 pseudovirions of Wuhan variant. IC50 concentrations of neutralizing mAbs were used for FCS binding studies. The concentrations of non-netralizing mAbs CR3022 and Covi-21 were 0.015 µg/ml. Autocorrelation plots were used to determine the binding efficiencies (% antibody bound) of a panel of A647 labeled anti-spike mAbs reacted with SARS-CoV-2 pseudovirions. (b) Relationships between Covi-series mAb binding efficiency measured with Wuhan pseudovirions were compared with their neutralization effect.

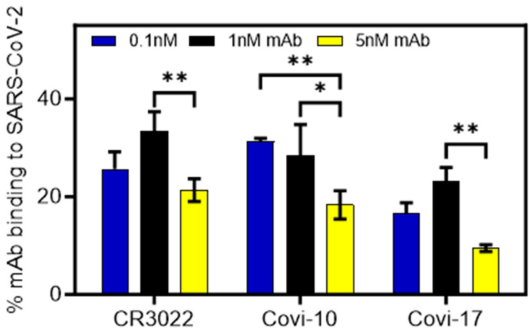

Figure S6. Binding of anti-spike mAbs to SARS-CoV-2 pseudovirions of Wuhan variant at the concentration range of 0.1 nM to 5nM. Autocorrelation plots were used to determine the binding efficiencies (% antibody bound) of A647 labeled anti-spike mAbs reacted with SARS-CoV-2 pseudovirions.

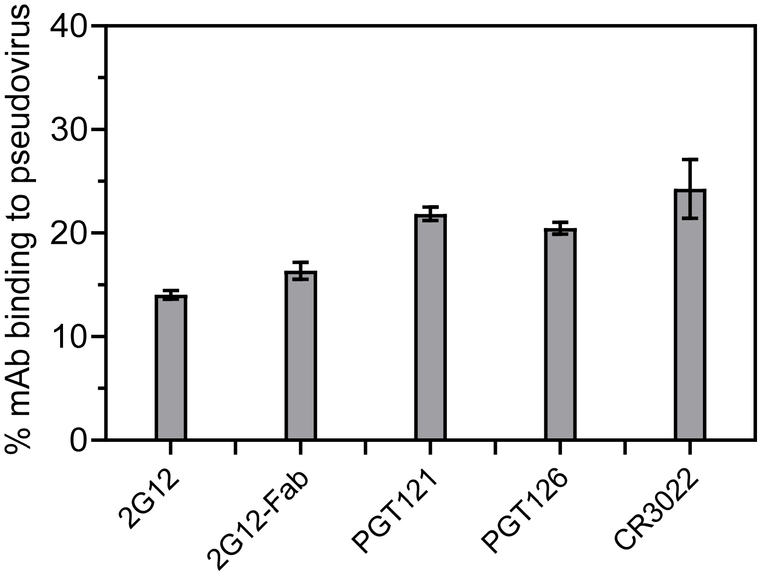

Figure S7. Comparative binding efficiencies of A647 labeled mAbs 2G12, PGT121, PGT126, CR3022 and Fab 2G12 with SARS-CoV-2 pseudovirion of Wuhan variant. All experiments were performed three times. Data are presented as the mean of three experiments ± SEM.

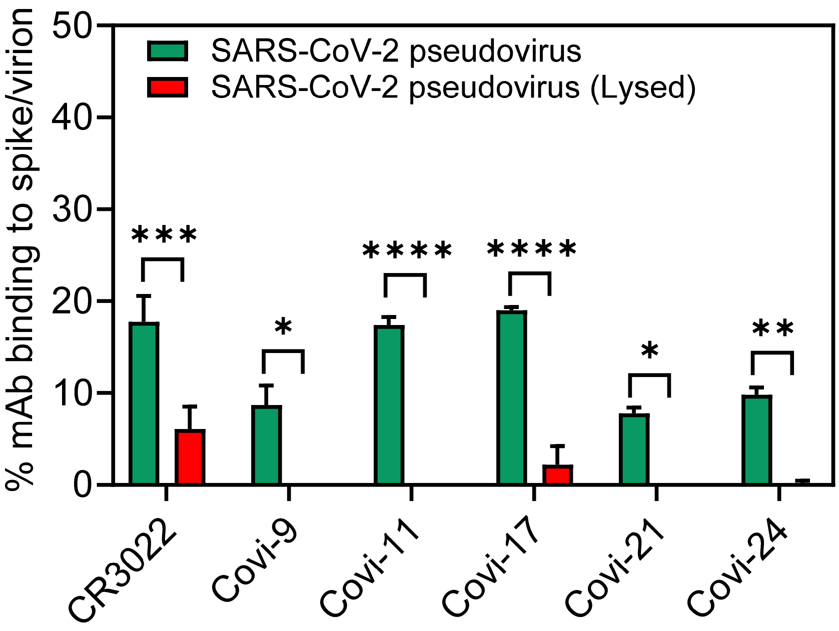

Figure S8. MAb CR3022 and Covi-series mAbs reactivity was further tested against spike proteins extant on whole pseudovirions or released from particles by detergent lysis. Diffusion coefficients were used to determine the spike protein disposition (virion-bound or released into solution; see text) reactive with the mAb. Autocorrelation plots and diffusion coefficients of signals were used to determine (see text) the efficiencies of CR3022 and Covi-series mAbs binding to pseudovirions or lysed virions. All experiments were performed three times. Data are presented as the mean of three experiments ± SEM. * ($p < 0.05$), ** ($p < 0.01$), *** ($p < 0.001$), and **** ($p < 0.0001$).
